# Heterologous Gene Expression in *Chlamydomonas reinhardtii* Chloroplast by Heterologous Promoters and Terminators, Intercistronic Expression Elements and Minichromosome

**DOI:** 10.1111/1751-7915.70069

**Published:** 2024-12-17

**Authors:** Yunling Guo, Hui Xiong, Qiuling Fan, Deqiang Duanmu

**Affiliations:** ^1^ State Key Laboratory of Agricultural Microbiology, Hubei Hongshan Laboratory Huazhong Agricultural University Wuhan China; ^2^ College of Life Science and Technology Huazhong Agricultural University Wuhan China; ^3^ Shenzhen Branch, Guangdong Laboratory for Lingnan Modern Agriculture, Genome Analysis Laboratory of the Ministry of Agriculture, Agricultural Genomics Institute at Shenzhen Chinese Academy of Agricultural Sciences Shenzhen China

**Keywords:** *Chlamydomonas reinhardtii*, chloroplast biotechnology, intercistronic expression elements, minichromosome, photosynthetic chassis

## Abstract

*Chlamydomonas reinhardtii* , a model green alga for expressing foreign proteins, faces challenges in multigene expression and enhancing protein expression level in the chloroplast. To address these challenges, we compared heterologous promoters, terminators and intercistronic expression elements (IEEs). We transformed Chlamydomonas chloroplast with a biolistic approach to introduce vectors containing the NanoLuc expression unit regulated by Chlamydomonas or tobacco promoters and terminators. We observed that tobacco promoters P*rbcL* and P*psbA* could not effectively regulate protein expression, whereas tobacco terminators T*rbcL* and T*rps16* did not affect the expression of Nluc protein. Further exploration of IEEs specific to Chlamydomonas revealed that Cr‐IEE2 had a minor effect on both upstream and downstream protein expression, whereas Cr‐IEE5 significantly influenced downstream protein expression. In contrast, tobacco IEE was found to be unsuitable for driving protein expression in Chlamydomonas. Additionally, VOR element and Rep protein derived from beet curly top geminivirus were able to form a minichromosome in Chlamydomonas chloroplast, and this system could enhance protein expression level compared to the traditional method of site‐specific integration in the plastome. This study highlights the potential of IEEs and minichromosome in facilitating heterologous protein expression in Chlamydomonas chloroplast.

## Graphical abstract

This study compared representative tobacco and Chlamydomonas promoters/terminators, intercistronic expression elements (IEEs), formation of a minichromosome and their effects on the heterologous protein expression in Chlamydomonas chloroplast. Results showed that the tobacco promoters and IEE do not function in Chlamydomonas, and VOR‐Rep‐mediated minichromosome could dramatically enhance protein expression levels in the algal chloroplast.

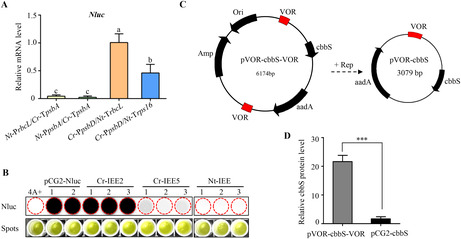

## 1 Introduction

Microalgae are recognised as valuable biotechnological platforms for the synthesis of high‐value compounds and recombinant proteins. *Chlamydomonas reinhardtii* , with the fully sequenced nuclear and chloroplast genomes and sophisticated genetic transformation tools, is considered an ideal photosynthetic chassis for expressing heterologous proteins (Salome and Merchant 2019). Notably, Chlamydomonas was the first organism being used for chloroplast transformation with the biolistic method (Boynton et al. 1988), which laid the foundation for chloroplast transformation in plants. Many valuable proteins, such as the synthetic human growth hormones, vaccines and other biopharmaceuticals, have been successfully expressed in Chlamydomonas chloroplast (Wannathong et al. 2016; Siddiqui et al. 2020). However, progress in developing multigene expression system in Chlamydomonas has been notably slower than that in tobacco. The nine α‐carboxysome‐related genes have been successfully integrated into tobacco chloroplast genome, whereas only one or two genes were typically expressed in Chlamydomonas chloroplast genome (Jackson et al. 2022; Chen et al. 2023).

To achieve efficient multigene expression, various regulatory components such as promoters, 5′ and 3′ untranslated regions (UTRs), terminators, intercistronic expression elements (IEEs), and selectable markers must be taken into account. In Chlamydomonas, many endogenous promoters and 5′UTRs have been characterised, including *16S*‐*atpA* (fusion of the *16S rRNA* gene promoter and 5′UTR of the *atpA* gene), *psbA*, *psbD*, *atpA*, *psaA* and *petB* (Bertalan et al. 2015; Larrea‐Alvarez and Purton 2020). A recent study showed that fusion of the 16S *rrn* promoter with the *rps4* 5′UTR significantly enhanced the rCry11A protein accumulation level when compared to using promoters and 5′UTRs of *psbD* and *atpA* genes (Odom et al. 2022). Limited studies have been conducted on the use of heterologous promoter elements in the chloroplast of Chlamydomonas. Previous studies demonstrated that promoters and 5′UTRs from other algal species, such as *atpA*, *tufA* and *psbD*, could regulate the transcription but not protein expression of the *psbA* gene in Chlamydomonas (Gimpel and Mayfield 2013). Terminators were found to have a minimal impact on protein accumulation in both Chlamydomonas and tobacco chloroplasts (Barnes et al. 2005; Tangphatsornruang et al. 2011). When using the same terminator for the expression of multiple foreign genes in Chlamydomonas chloroplast, partial gene loss may occur (Larrea‐Alvarez and Purton 2020). The repeated use of endogenous elements could also lead to homologous recombination, potentially causing genetic instability of chloroplast genome. Therefore, there is a greater need to expand the pool of heterologous promoter and terminator elements for Chlamydomonas chloroplast biotechnology.

In addition to promoters and terminators, the expression of multigenes in monocistronic or polycistronic forms is a crucial factor. Previous studies identified at least 16 clusters of transcriptional units in Chlamydomonas chloroplast (Gallaher et al. 2018), suggesting the presence of potential operons for multigene expression in this organism. Polycistronic mRNA can be efficiently translated in tobacco chloroplast (Staub and Maliga 1995). Small IEEs of 50–100 base pairs have been discovered in tobacco, playing a key role in cleaving polycistronic mRNA into monocistronic units, which enables the efficient translation of downstream genes (Zhou, Karcher, and Bock 2007; Legen et al. 2018). These IEEs have also been utilised to express the metabolic pathways of vitamin E in both tobacco and tomato plants (Lu et al. 2013). In Chlamydomonas, two functional IEEs were shown to be able to regulate the expression of downstream GFP protein (Macedo‐Osorio et al. 2018). The discovery of IEEs provided new tools for multigene expression in chloroplast.

In recent years, research has shown that foreign genes can be expressed in the form of minichromosomes in tobacco (Jakubiec et al. 2021). This system relies on geminivirus replication initiation protein (Rep) and viral replication initiation sequence (VOR). Geminivirus belongs to a large family of plant viruses with a broad host range, leading to the production of many replicators through the rolling‐circle replication (Wang et al. 2017). Plasmid‐like DNA molecules have also been discovered in some algal species. In dinoflagellate chloroplasts, around 20 plasmid‐like DNA molecules are present, each containing core regions with replication initiation sequences and transcription initiation sites. Introduction of artificial minicircle plasmids into dinoflagellates results in transformants that can be maintained for up to a year (Nimmo et al. 2019). Furthermore, minicircles have been found in the Stylonematophyceae red algae, containing specific segments of the mitochondrial genome (Lee et al. 2023). Those results suggest that minichromosomes could potentially be used as a genetic tool to enhance the expression level of proteins in algae.

This study aims to broaden the heterologous elements in Chlamydomonas chloroplast by investigating the regulation of NanoLuc (Nluc) protein expression using tobacco promoters or terminators, tobacco and Chlamydomonas IEEs, and minichromosome. Results showed that tobacco promoters were ineffective, and the tobacco terminators were compatible with Chlamydomonas promoters in controlling protein expression. Furthermore, the study demonstrated that the Cr‐IEE2 element has a minor impact on the expression levels of the upstream cbbL and downstream Nluc proteins, whereas Cr‐IEE5 significantly affects the expression level of downstream Nluc protein in the multigene expression of Chlamydomonas. The minichromosome could be present in Chlamydomonas chloroplast and enhanced the protein expression level. These findings underscore the applicability of these tools for facilitating multigene expression in Chlamydomonas chloroplast.

## 2 Results and Discussion

### 2.1 Comparison of Tobacco Promoters and Terminators in Regulating Protein Expression in Chlamydomonas Chloroplast

**FIGURE 1:**
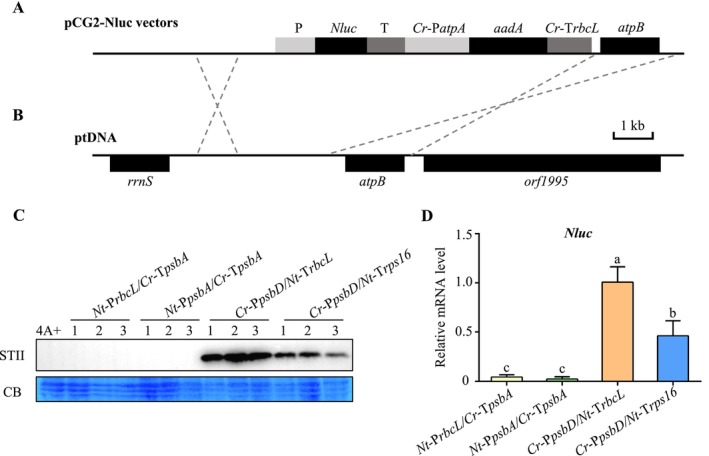
Comparison of NanoLuc protein expression using tobacco or Chlamydomonas promoters and terminators. (A) Construction of plastid transformation vectors (pCG2‐Nluc). The *Nluc* gene is placed under the control of distinct promoters and terminators, along with the spectinomycin‐resistance marker gene *aadA*. P, promoter; T, terminator; Cr and Nt indicate *Chlamydomonas reinhardtii* and *Nicotiana benthamiana*, respectively; *Cr*‐P*atpA*, promoter of *atpA gene* of *C. reinhardtii* ; *Cr*‐T*rbcL*, terminator of *rbcL* gene of *C. reinhardtii* . (B) Illustration of the targeting region in the Chlamydomonas plastid genome (ptDNA). The transgenes are integrated into the intergenic region between the *rrnS* and *atpB* genes. In (A) and (B), flanking sequences that enable homologous recombination in the Chlamydomonas plastid genome are marked by dashed lines. Scale bar, 1 kb. (C) Detection of Nluc protein accumulation in wild‐type 4A+ and transgenic *C. reinhardtii* strains. Immunoblot analysis of Nluc protein accumulation was performed using an antibody against the Strep tag II (STII), and Coomassie blue staining (CB) was used for protein equal loading. *Nt*‐P*rbcL/Cr*‐T*psbA* represents combination of the tobacco *rbcL* gene promoter and the Chlamydomonas *psbA* gene terminator. Other three types of promoter/terminator combinations include *Nt*‐P*psbA/Cr*‐T*psbA*, *Cr*‐P*psbD/Nt*‐T*rbcL* and *Cr*‐P*psbD/Nt*‐T*rps16*. For each transformation, three independent transgenic strains were randomly selected for analysis. (D) Quantification of the expression levels of *Nluc* transcript in transgenic Chlamydomonas strains by RT‐qPCR. The *CBLP* gene (Cre06.g278222) was used as the endogenous control, and error bars represent the standard deviation calculated from three biological replicates. Different lowercase letters indicate significant differences based on Tukey's multiple comparisons test (*p* < 0.05).

We investigated the feasibility of using tobacco promoters/5′UTRs or terminators/3′UTRs to express the Nluc protein in Chlamydomonas. Three promoters (*Nt*‐P*rbcL*, *Nt*‐P*psbA*, *Nt*‐P*psbD*) and two terminators (*Nt*‐T*rbcL*, *Nt*‐T*rps16*) from *Nicotiana benthamiana* were selected. Four pCG2 constructs were generated, each containing the Nluc coding region with the Strep Tag II (STII, WSHPQFEK). One set included the *Nt*‐P*rbcL* or *Nt*‐P*psbA* promoters with the Chlamydomonas *psbA* terminator (*Cr*‐T*psbA*). The other set consisted of the *Cr*‐P*psbD* promoter with tobacco terminators, *Nt*‐T*rbcL* or *Nt*‐T*rps16* (Figure 1A). The pCG2 vector contains homologous sequences so that the Nluc expression cassette is inserted into specific sites between the *rrnS* and *atpB* genes through homologous recombination in Chlamydomonas chloroplast (Figure 1B).

The four vectors were individually transformed into wild‐type *C. reinhardtii* 4A+ chloroplasts by biolistic transformation. The transformants were selected in the presence of 100 μg/mL spectinomycin. Immunoblot analysis was performed to compare Nluc protein expression by utilising Strep tag II antibody. The results showed that none of the transgenic strains exhibited Nluc protein accumulation with tobacco promoters P*rbcL*, P*psbA* or P*psbD* (Figure 1C, Figure S1). In contrast, the Chlamydomonas promoter P*psbD* in combination with the tobacco terminators T*rbcL* or T*rps16* could result in the accumulation of Nluc protein, and the Nluc protein level is higher when the Chlamydomonas P*psbD* promoter is paired with the tobacco terminator T*rbcL* relative to T*rps16* (Figure 1C). Consistently, the Chlamydomonas P*psbD* promoter resulted in much higher Nluc mRNA levels than the tobacco promoters P*rbcL* and P*psbA* (Figure 1D). Those data suggest that regulatory elements within the promoter play a more significant role in controlling gene expression efficiency in Chlamydomonas chloroplast.

It has been reported that the promoter and terminator elements of Chlamydomonas can be utilised in tobacco (Fuentes et al. 2016; Long et al. 2018). The discrepancy indicates potential differences in transcriptional and translational mechanisms between Chlamydomonas and tobacco. Chlamydomonas chloroplasts possess a single plastid‐encoded RNA polymerase, whereas higher plants typically have two sets of RNA polymerases, plastid‐encoded and nuclear‐encoded (Yagi and Shiina 2014). Sequence alignments of the *rbcL*, *psbA* and *psbD* promoters showed that Chlamydomonas and tobacco promoters exhibited lower identities, ranging from 17.71% to 37.34% (Figure S2). In Chlamydomonas, regulation of chloroplast gene expression largely occurs at the post‐transcriptional level. Studies have indicated that the 5′UTR of Chlamydomonas chloroplast genes has species‐specific characteristics (Gimpel and Mayfield 2013), whereas terminators typically have minimal influence on translational regulation. Our data showed that tobacco terminators could be used in Chlamydomonas, thereby expanding the repertoire of gene expression elements in this model alga.

### 2.2 Cr‐IEE2 Effectively Regulates Downstream Protein Expression Compared to Cr‐IEE5, Whereas the Tobacco IEE Is Inefficient in Chlamydomonas

**FIGURE 2:**
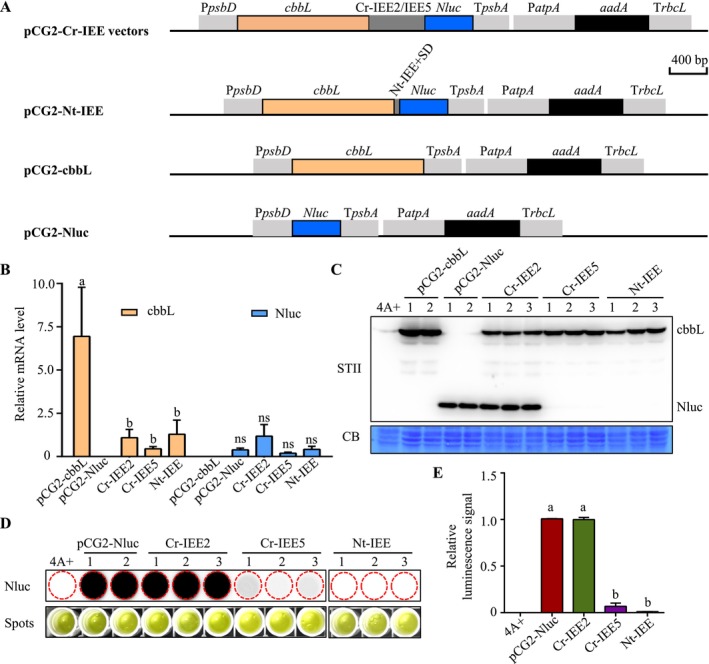
Comparison of Nluc protein expression using tobacco or Chlamydomonas intercistronic expression elements (IEEs). (A) Illustration of chloroplast transformation vectors. In pCG2‐Cr‐IEE vectors, the expression cassettes *cbbL*/Cr‐IEEs/*Nluc* are regulated by the Chlamydomonas *psbD* promoter and *psbA* terminator. In pCG2‐Nt‐IEE vector, *cbbL*/Nt‐IEE/*Nluc* is also controlled by the *psbD* promoter and *psbA* terminator. IEE, intercistronic expression element; SD, Shine‐Dalgarno sequence. Scale bar, 400 bp. (B) Detection of the relative expression levels of *cbbL* and *Nluc* transcripts in transgenic Chlamydomonas strains by RT‐qPCR. The Chlamydomonas *CBLP* gene was used as the endogenous control, and the *cbbL*/*Nluc* mRNA levels were compared to Cr‐IEE2. Error bars represent the standard deviation calculated from three biological replicates. Different letters show significant differences based on Tukey's multiple comparisons test (*p* < 0.05). ns, not significant. (C) Immunoblot detection of cbbL and Nluc protein accumulation by using the STII antibody, with Coomassie blue (CB) staining as an equal loading control. (D) Detection of in vivo luciferase activity in the transgenic strains. (E) Relative quantification of the luminescence signals in wild‐type 4A+ and transgenic strains. Signal intensities were compared to Cr‐IEE2. Error bars represent the standard deviation calculated from three biological replicates. Different letters show significant differences based on Tukey's multiple comparisons test (*p* < 0.05).

In Chlamydomonas chloroplasts, two intercistronic expression elements (IEEs) were successfully used for the expression of *aphA‐6* and *GFP* genes (Macedo‐Osorio et al. 2018). However, the application of IEEs for multigene expression in Chlamydomonas chloroplasts is limited. Our study aims to investigate the impact of these IEEs on the expression levels of both upstream and downstream proteins. The synthetic operons Cr‐IEE2 and Cr‐IEE5 containing the *cbbL* (encoding the Rubisco large subunit of *Thiobacillus neapolitanus* ) and *Nluc* (encoding NanoLuc), along with the selectable marker gene *aadA*, were constructed into the pCG2 vector (Figure 2A). After achieving homoplasmy, qPCR was used to quantify the mRNA levels of the upstream *cbbL* and downstream *Nluc* genes. We found that, compared to the pCG2‐cbbL lines, the mRNA levels of the upstream *cbbL* were reduced in the Cr‐IEE2 and Cr‐IEE5 lines. However, there was no significant difference in the mRNA levels of the downstream Nluc among the Cr‐IEE2, Cr‐IEE5 and the pCG2‐Nluc lines (Figure 2B). The accumulation of the cbbL protein was slightly lower in the Cr‐IEE2 and Cr‐IEE5 lines compared to the pCG2‐cbbL lines (Figure 2C). Those data suggests that the expression of upstream protein is partially affected by the Chlamydomonas IEEs. The accumulation of the Nluc protein showed no significant difference in the Cr‐IEE2 lines compared to the pCG2‐Nluc lines, whereas it was undetectable in the Cr‐IEE5 lines (Figure 2C). A very weak luminescence signal for Nluc luciferase was detected in the Cr‐IEE5 lines (Figure 2D). The relative luminescence signal intensity of Cr‐IEE2 was approximately 15‐folds higher than that of Cr‐IEE5 (Figure 2E). These results indicate that Cr‐IEE2, when compared to Cr‐IEE5, showed better performance in regulating downstream protein expression. Previous study observed that both Cr‐IEE2 and Cr‐IEE5 exhibited similar capacities in controlling the expression of downstream GFP protein (Macedo‐Osorio et al. 2018). The discrepancy could be attributable to variations in downstream gene sequences and post‐transcriptional regulation. Chlamydomonas IEE2 and IEE5 have a sequence of 400–600 bp (Macedo‐Osorio et al. 2018). Due to their excessive length, they are not suitable for repeated use in multigene expression, as homologous recombination may occur between the same IEEs. Therefore, investigating the shorter Chlamydomonas IEE elements has the potential to broaden their applications in multigene expression engineering.

Tobacco IEE elements have been commonly used for multigene expression in tobacco and tomato plastids, but their application in Chlamydomonas is not well understood. We designed the synthetic operon Nt‐IEE that consisted of *cbbL* and *Nluc* genes. Translation initiation of the downstream gene was enabled by the Shine‐Dalgarno sequence of the tobacco *RbcL* gene (Figure 2A). Transformants with tobacco IEE were tested using colony PCR, followed by the assessment of Nluc luciferase activity. However, protein expression and luminescence signal were not detectable in the Nt‐IEE transplastomic lines compared to that in the positive control pCG2‐Nluc (Figure 2C,D). These data suggest that tobacco IEE element may not be suitable for gene expression in Chlamydomonas. There could be two possible reasons. First, the absence of the half‐a‐tetratricopeptide (HAT) repeat protein HCF107, which is essential for recognising and binding tobacco IEE sequences, may result in ineffective mRNA protection and translational regulation in Chlamydomonas (Hammani, Cook, and Barkan 2012). Second, the tobacco SD sequence may not be efficiently recognised for translation initiation in Chlamydomonas.

In higher plants, most chloroplast genes are organised into operons, necessitating polycistrons to be processed into monocistrons for efficient translation (Zhou, Karcher, and Bock 2007). Using IEE elements for multigene expression has the benefit of reducing the number of promoters required, thereby decreasing the vector size and improving transformation efficiency. Research has shown that four additional IEE‐like elements can also regulate the translation of downstream proteins in tobacco (Legen et al. 2018). Therefore, it should be useful to systemically compare candidate IEE elements for multigene expression in Chlamydomonas.

### 2.3 The VOR Sequence Undergoes Recombination to Form Minichromosome

**FIGURE 3:**
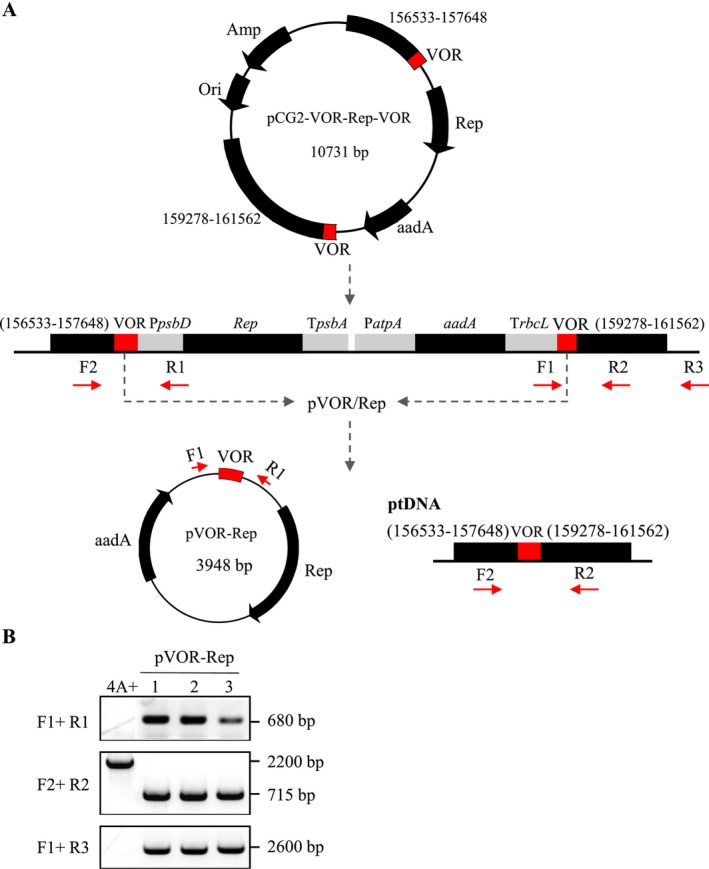
Minichromosome formation in Chlamydomonas chloroplast. (A) Schematic diagram of pCG2‐VOR‐Rep‐VOR vector. Two VOR sequences are expected to recombine and form an extrachromosomal circular plasmid, designated as a minichromosome (pVOR‐Rep, 3948 bp). VOR, viral origin of replication; Rep, replication initiator protein. (B) PCR identification of the minichromosome (pVOR‐Rep) in transgenic *C. reinhardtii* strains using F1 and R1 primers. Confirmation of VOR scars at the insertion site of chloroplast genome was performed by PCR with F2 and R2 primers. The F1 and R3 primers were used to confirm that pCG2‐VOR‐Rep‐VOR was still present in the plastome.

A replicating minichromosome system has been successfully developed for chloroplast gene expression in tobacco (Jakubiec et al. 2021). However, this system has not yet been reported in Chlamydomonas chloroplast. We then aimed to investigate the potential formation of minichromosomes in Chlamydomonas chloroplast by generating a plastid transformation vector encoding the *Rep* and *aadA* genes that are controlled by Chlamydomonas promoters. The vector pCG2‐VOR‐Rep‐VOR contained two VOR sequences flanking the expression cassette, with homologous arms targeting the plastid genome‐specific regions (Figure 3A). Circular minichromosome, named pVOR‐Rep, was successfully formed and confirmed by PCR using site‐specific primers (F1 and R1) that were oriented in opposite directions (Figure 3). A VOR sequence remained as a scar in the genome of the transplastomic lines, as detected by PCR using another set of primers (F2 and R2). Additionally, some transgenes were found to remain integrated in the genome (primers F1 and R3). As the length of the homologous arm sequences increased, the transformation efficiency also increased (Dauvillee et al. 2004). Although shorter sequences can undergo recombination, it is hypothesised that in the absence of selective pressure, sequences shorter than 200 bp may not be fully recombined from the Chlamydomonas genome (Jackson et al. 2022). Thus, with the VOR sequence being 201 bp long, partial recombination between the two VOR sequences may result in the formation of minichromosomes.

### 2.4 The Minichromosome Enhanced Protein Expression in Chlamydomonas

**FIGURE 4:**
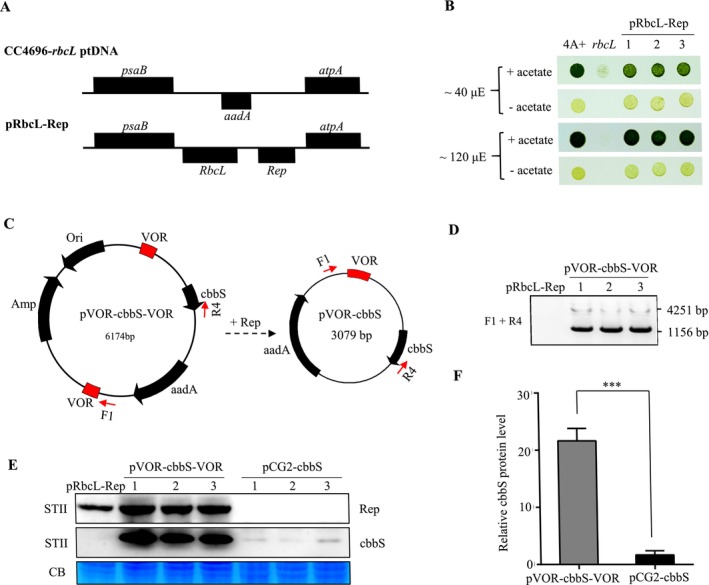
Minichromosome enhanced expression levels of the heterologous cbbS protein. (A) Schematic diagram of the target region for RbcL and Rep expression cassettes in the plastome of the Chlamydomonas CC4696‐*rbcL*∆ mutant. (B) Mixotrophic (+ acetate) and photoautotrophic (− acetate) growth of 4A+, *rbcL* mutant, and pRbcL‐Rep rescued strains under different light intensities. (C) Schematic diagram of the pVOR‐cbbS‐VOR vector used for chloroplast transformation and formation of the minichromosome (pVOR‐cbbS) in transgenic strains. (D) PCR identification of the pVOR‐cbbS minichromosome with the primers F1 and R4 as depicted in (C). (E) Immunoblot analysis of cbbS and Rep proteins in pVOR‐cbbS‐VOR transformants by using STII antibody. The pCG2‐cbbS transgenic strains were used as control. The same cbbS expression cassette (Chlamydomonas *atpA* promoter and *psbA* terminator) was used in pVOR‐cbbS‐VOR and pCG2‐cbbS. CB, Coomassie blue staining. (F) Accumulation levels of cbbS protein in pVOR‐cbbS‐VOR and pCG2‐cbbS transgenic strains. Relative band intensities in immunoblots were quantified using ImageJ and error bars represent the standard deviation (SD) of three replicates. Asterisk denotes significant difference (*p* < 0.001) as determined by the Student's *t*‐test.

The above results indicate that minichromosomes can indeed form in Chlamydomonas. We then analysed whether the minichromosome system could enhance the production of heterologous proteins, similar to what has been observed in tobacco (Jakubiec et al. 2021). We first generated pRbcL‐Rep transplastomic lines in the CC‐4696 rbcL∆‐MX3312 background, in which RbcL and Rep expression cassettes were integrated between *psaB* and *atpA* genes (Figure 4A). Screening of pRbcL‐Rep transplastomic lines was conducted on TP (Tris‐phosphate) plates without acetate, and the restoration of the photosynthetic growth phenotype was confirmed by spot test (Figure 4B). One such pRbcL‐Rep transplastomic line was then transformed with the pVOR‐cbbS‐VOR vector, which carries the *cbbS* and *aadA* genes controlled by Chlamydomonas *atpA* and *psaA‐1* promoters, respectively (Figure 4C). The *cbbS* gene encoding the Rubisco small subunit of *Thiobacillus neapolitanus* was codon optimised based on the chloroplast codon usage frequency of *Chlamydomonas reinhardtii* . Notably, the pVOR‐cbbS‐VOR vector lacks homologous arms and therefore does not integrate into the plastid genome. PCR analysis confirmed the expected size, with a 1156 bp band indicating the recombination between two VOR sequences and the presence of circular minichromosomes (Figure 4D). Additionally, a larger but weaker band of 4251 bp was also observed, corresponding to the fragment of the original vector, pVOR‐cbbS‐VOR (Figure 4D). Western blot analysis confirmed the expression of Rep protein in the pVOR‐cbbS‐VOR transformants and showed dramatically enhanced accumulation of cbbS protein in all transplastomic lines compared to the pCG2‐cbbS lines, where *cbbS* expression cassette undergoes site‐specific integration into the plastome (Figure 4E). Quantitative analysis revealed that the accumulation of cbbS protein was more than 20‐folds higher in the pVOR‐cbbS‐VOR transplastomic lines compared to the pCG2‐cbbS lines (Figure 4F). Furthermore, immunoblot analysis showed that the accumulation levels of endogenous RbcL and RbcS proteins were not affected, and no significant growth differences were observed in those transplastomic lines (Figure S3). Collectively, those findings indicate that minichromosomes are valuable tools for high‐level expression of heterologous genes in Chlamydomonas chloroplast.

The minichromosome system of geminivirus was utilised to express GFP protein in tobacco chloroplasts, resulting in a four‐fold increase in protein accumulation (Jakubiec et al. 2021). Moreover, geminivirus vectors have been utilised as transient expression tools in the nucleus of Chlamydomonas for expressing foreign proteins (Malla et al. 2021). One advantage of the minichromosome system is that it does not integrate into the chloroplast genome and should theoretically have minimal impact on the expression of endogenous genes. However, the geminivirus system is constrained by its cargo capacity limitation. If the DNA fragment of the vector is too large (i.e., > 7 kb), it may inhibit replication of the vector in vivo and therefore reduce the expression level of foreign proteins (Wang et al. 2017). Overall, geminivirus vector systems exhibit great potential for a wide range of biotechnological applications.

## 3 Conclusions

Chlamydomonas has well‐established chloroplast and nuclear transformation systems for expressing valuable proteins. In the era of synthetic biology, there is a growing need for high‐throughput detection and comparison of both endogenous and heterologous promoter elements to improve gene expression levels in Chlamydomonas. The identification and design of IEEs are essential for enabling multigene expression in a polycistronic manner. Minichromosomes exist autonomously in Chlamydomonas and protein expression level was significantly enhanced compared to the traditional method of homologous integration into specific sites of plastome. These tools have potential in reconstruction of complex metabolic pathways for the production of high‐value products in Chlamydomonas chloroplasts.

## Author Contributions

**Yunling Guo:** conceptualization, methodology, investigation, validation, writing – original draft, writing – review and editing, formal analysis. **Hui Xiong:** conceptualization, investigation, methodology, validation, writing – original draft. **Qiuling Fan:** conceptualization, writing – review and editing. **Deqiang Duanmu:** conceptualization, funding acquisition, project administration, supervision, writing – original draft, writing – review and editing.

## Conflicts of Interest

The authors declare no conflicts of interest.

## Supporting information


**FIGURE S1.**. Comparison of NanoLuc protein expression using different promoters and terminators. (A) Detection of Nluc protein accumulation in transgenic *C. reinhardtii* strains using a combination of *Nicotiana benthamiana* or *Chlamydomonas reinhardtii* promoters with the same Chlamydomonas *psbA* terminator. Immunoblot analysis of Nluc protein accumulation was performed using an antibody against the Strep tag II (STII), and Coomassie blue staining (CB) was used for protein equal loading. *Cr*‐P*rbcL/Cr*‐T*psbA* represents a combination of the Chlamydomonas *rbcL* gene promoter and the Chlamydomonas *psbA* gene terminator. Other three types of promoter/terminator combinations include *Cr*‐P*psbA/Cr*‐T*psbA*, *Nt*‐P*rbcL/Cr*‐T*psbA*, and *Nt*‐P*psbA/Cr*‐T*psbA*. For each transformation, three independent transgenic strains were selected for analysis. (B) Detection of Nluc protein accumulation in transgenic *C. reinhardtii* strains using a combination of tobacco or Chlamydomonas *psbD* promoters with tobacco terminators. The promoter/terminator combinations include *Cr*‐P*psbD/Nt*‐T*rbcL*, *Cr*‐P*psbD/Nt*‐T*rps16*, *Nt*‐P*psbD/Nt*‐T*rbcL*, and *Nt*‐P*psbD/Nt*‐T*rps16*.
**FIGURE S2.**. Sequence alignments of the representative Chlamydomonas and tobacco promoters. Sequence alignments of the *rbcL* promoters (A), *psbA* promoters (B), and *psbD* promoters (C) from *Chlamydomonas reinhardtii* and *Nicotiana benthamiana* were respectively performed and the identity levels were indicated.
**FIGURE S3.**. Expression of cbbS does not affect accumulation of the endogenous RbcL and RbcS proteins. (A) Detection of RbcL and RbcS protein accumulation in pRbcL‐Rep, pVOR‐cbbS‐VOR, and pCG2‐cbbS transformants. Coomassie blue staining (CB) was used for protein equal loading. (B) Mixotrophic (+ acetate) and photoautotrophic (− acetate) growth comparison of pRbcL‐Rep, pVOR‐cbbS‐VOR and pCG2‐cbbS strains in continuous light (40 or 120 μE). Equal numbers of cells (~3000) were spotted on the plates, and images were captured after 5–7 days of growth.

**TABLE S1.** Primers used in this study.

**Data S1.**.

## Data Availability

The data that support the findings of this study are available from the corresponding author upon reasonable request.
