# Supplementary material for: Heterologous Gene Expression in Chlamydomonas reinhardtii Chloroplast by Heterologous Promoters and Terminators, Intercistronic Expression Elements and Minichromosome
Source: Microb Biotechnol. 2024 Dec 17;17(12):e70069. doi: 10.1111/1751-7915.70069 (PMC11650887; doi:10.1111/1751-7915.70069)
Supplement: Supplementary file 1 — FIGURE S1.. Comparison of NanoLuc protein expression using different promoters and terminators. (A) Detection of Nluc protein accumulation in transgenic C. reinhardtii strains using a combination of Nicotiana benthamiana or Chlamydomonas reinhardtii promoters with the same Chlamydomonas psbA terminator. Immunoblot analysis of Nluc protein accumulation was performed using an antibody against the Strep tag II (STII), and Coomassie blue staining (CB) was used for protein equal loading. Cr‐PrbcL/Cr‐TpsbA represents a combination of the Chlamydomonas rbcL gene promoter and the Chlamydomonas psbA gene terminator. Other three types of promoter/terminator combinations include Cr‐PpsbA/Cr‐TpsbA, Nt‐PrbcL/Cr‐TpsbA, and Nt‐PpsbA/Cr‐TpsbA. For each transformation, three independent transgenic strains were selected for analysis. (B) Detection of Nluc protein accumulation in transgenic C. reinhardtii strains using a combination of tobacco or Chlamydomonas psbD promoters with tobacco terminators. The promoter/terminator combinations include Cr‐PpsbD/Nt‐TrbcL, Cr‐PpsbD/Nt‐Trps16, Nt‐PpsbD/Nt‐TrbcL, and Nt‐PpsbD/Nt‐Trps16. FIGURE S2.. Sequence alignments of the representative Chlamydomonas and tobacco promoters. Sequence alignments of the rbcL promoters (A), psbA promoters (B), and psbD promoters (C) from Chlamydomonas reinhardtii and Nicotiana benthamiana were respectively performed and the identity levels were indicated. FIGURE S3.. Expression of cbbS does not affect accumulation of the endogenous RbcL and RbcS proteins. (A) Detection of RbcL and RbcS protein accumulation in pRbcL‐Rep, pVOR‐cbbS‐VOR, and pCG2‐cbbS transformants. Coomassie blue staining (CB) was used for protein equal loading. (B) Mixotrophic (+ acetate) and photoautotrophic (− acetate) growth comparison of pRbcL‐Rep, pVOR‐cbbS‐VOR and pCG2‐cbbS strains in continuous light (40 or 120 μE). Equal numbers of cells (~3000) were spotted on the plates, and images were captured after 5–7 days of growth. [file MBT2-17-e70069-s003.pptx]

## Slide 1
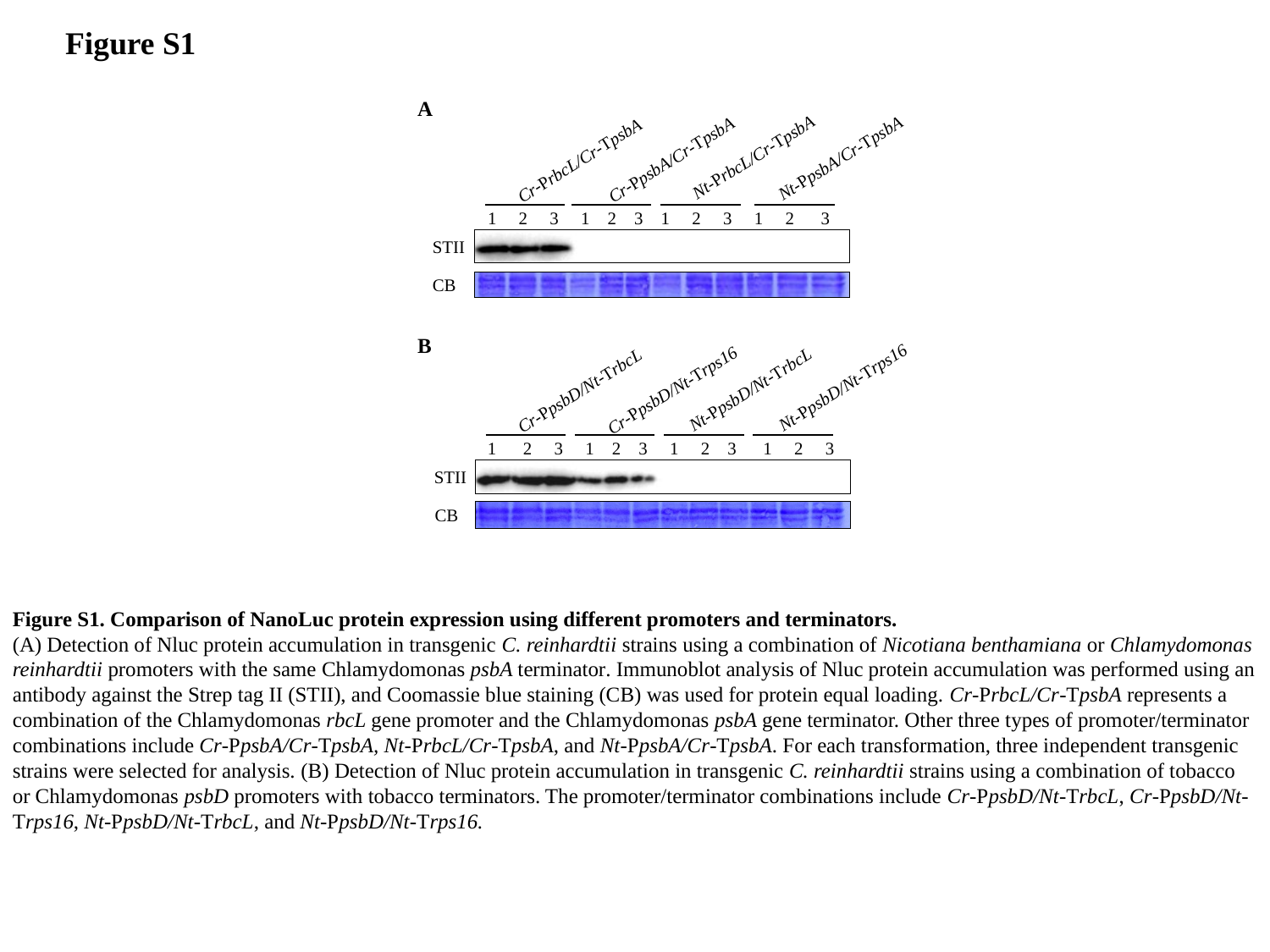

Figure S1
A
Nt-PrbcL/Cr-TpsbA
Cr-PrbcL/Cr-TpsbA
Cr-PpsbA/Cr-TpsbA
Nt-PpsbA/Cr-TpsbA
 1 2 3 1 2 3 1 2 3 1 2 3
STII
CB
B
Nt-PpsbD/Nt-TrbcL
Cr-PpsbD/Nt-TrbcL
Cr-PpsbD/Nt-Trps16
Nt-PpsbD/Nt-Trps16
 1 2 3 1 2 3 1 2 3 1 2 3
STII
CB
Figure S1. Comparison of NanoLuc protein expression using different promoters and terminators.
(A) Detection of Nluc protein accumulation in transgenic C. reinhardtii strains using a combination of Nicotiana benthamiana or Chlamydomonas reinhardtii promoters with the same Chlamydomonas psbA terminator. Immunoblot analysis of Nluc protein accumulation was performed using an antibody against the Strep tag II (STII), and Coomassie blue staining (CB) was used for protein equal loading. Cr-PrbcL/Cr-TpsbA represents a combination of the Chlamydomonas rbcL gene promoter and the Chlamydomonas psbA gene terminator. Other three types of promoter/terminator combinations include Cr-PpsbA/Cr-TpsbA, Nt-PrbcL/Cr-TpsbA, and Nt-PpsbA/Cr-TpsbA. For each transformation, three independent transgenic strains were selected for analysis. (B) Detection of Nluc protein accumulation in transgenic C. reinhardtii strains using a combination of tobacco or Chlamydomonas psbD promoters with tobacco terminators. The promoter/terminator combinations include Cr-PpsbD/Nt-TrbcL, Cr-PpsbD/Nt-Trps16, Nt-PpsbD/Nt-TrbcL, and Nt-PpsbD/Nt-Trps16.

## Slide 2
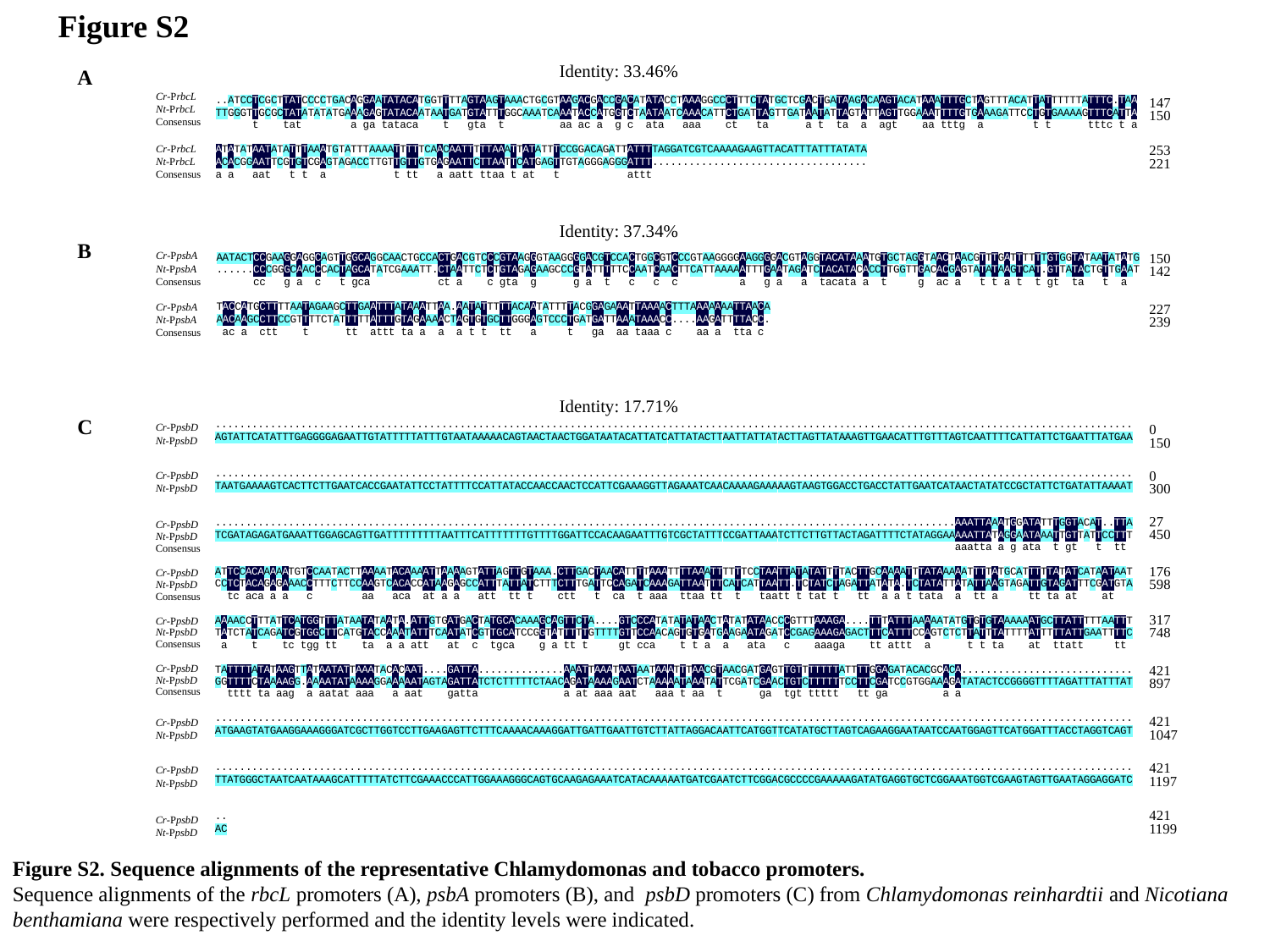

Figure S2
Identity: 33.46%
A
Cr-PrbcL
147
Nt-PrbcL
150
Consensus
253
Cr-PrbcL
221
Nt-PrbcL
Consensus
Identity: 37.34%
B
Cr-PpsbA
150
Nt-PpsbA
142
Consensus
Cr-PpsbA
227
Nt-PpsbA
239
Consensus
Identity: 17.71%
C
Cr-PpsbD
0
Nt-PpsbD
150
0
Cr-PpsbD
300
Nt-PpsbD
27
Cr-PpsbD
450
Nt-PpsbD
Consensus
176
Cr-PpsbD
598
Nt-PpsbD
Consensus
317
Cr-PpsbD
748
Nt-PpsbD
Consensus
421
Cr-PpsbD
Nt-PpsbD
897
Consensus
421
Cr-PpsbD
1047
Nt-PpsbD
421
Cr-PpsbD
1197
Nt-PpsbD
421
Cr-PpsbD
1199
Nt-PpsbD
Figure S2. Sequence alignments of the representative Chlamydomonas and tobacco promoters.
Sequence alignments of the rbcL promoters (A), psbA promoters (B), and psbD promoters (C) from Chlamydomonas reinhardtii and Nicotiana benthamiana were respectively performed and the identity levels were indicated.

## Slide 3
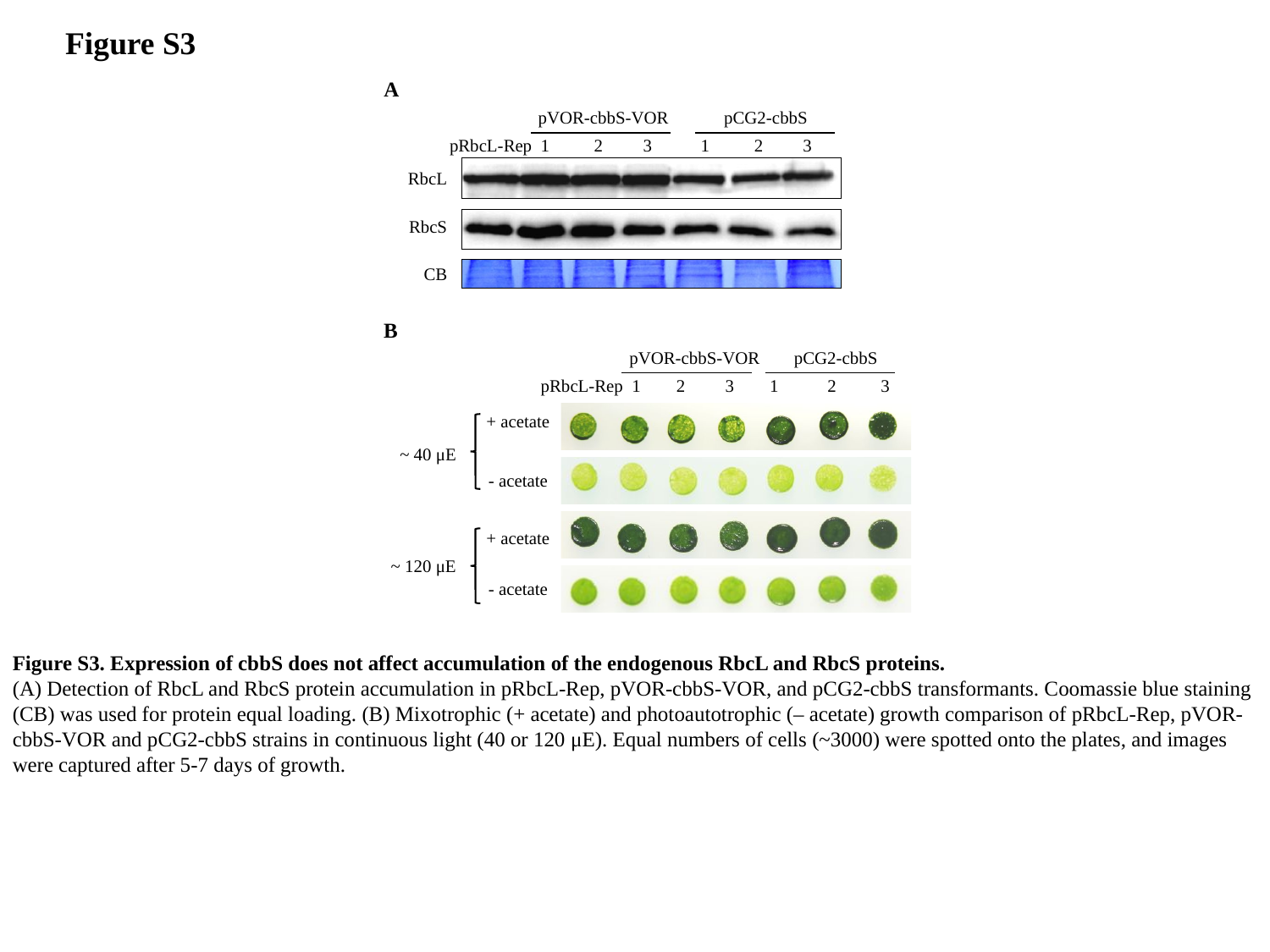

Figure S3
A
pVOR-cbbS-VOR
pCG2-cbbS
 pRbcL-Rep 1 2 3 1 2 3
RbcL
RbcS
CB
B
pVOR-cbbS-VOR
pCG2-cbbS
 pRbcL-Rep 1 2 3 1 2 3
+ acetate
~ 40 μE
- acetate
+ acetate
~ 120 μE
- acetate
Figure S3. Expression of cbbS does not affect accumulation of the endogenous RbcL and RbcS proteins.
(A) Detection of RbcL and RbcS protein accumulation in pRbcL-Rep, pVOR-cbbS-VOR, and pCG2-cbbS transformants. Coomassie blue staining (CB) was used for protein equal loading. (B) Mixotrophic (+ acetate) and photoautotrophic (– acetate) growth comparison of pRbcL-Rep, pVOR-cbbS-VOR and pCG2-cbbS strains in continuous light (40 or 120 μE). Equal numbers of cells (~3000) were spotted onto the plates, and images were captured after 5-7 days of growth.
