# Supplementary material for: Heterologous Gene Expression in Chlamydomonas reinhardtii Chloroplast by Heterologous Promoters and Terminators, Intercistronic Expression Elements and Minichromosome
Source: Microb Biotechnol. 2024 Dec 17;17(12):e70069. doi: 10.1111/1751-7915.70069 (PMC11650887; doi:10.1111/1751-7915.70069)
Supplement: Supplementary file 3 — Data S1.. [file MBT2-17-e70069-s002.docx]

**Experimental Procedures**

**Chlamydomonas strains and growth conditions**

*Chlamydomonas reinhardtii* strain 4A+ (mating type plus) was used as the wild-type recipient strain for chloroplast transformation. The CC-4696 rbcL∆-MX3312 mt+ strain was obtained from the Chlamydomonas Resource Center, and was used as the host for chloroplast transformation. All Chlamydomonas strains were maintained on TAP plates at 24^0^C under a 20-hour dark/4-hour light cycle. For both chloroplast transformation and total protein extraction, Chlamydomonas cells were grown in TAP liquid medium under continuous low light condition (~ 30 μmol photons m^-2^ s^-1^).

**Plasmid construction**

The tobacco promoters (*Nt*-P*rbcL*, *Nt*-P*psbA* and *Nt*-P*psbD*) and terminators (*Nt*-T*rbcL* and *Nt*-T*rps16*) were amplified from the tobacco genomic DNA. The promoter *Cr-*P*psbD*, *Cr-*P*rbcL*, *Cr-*P*psbA* and terminator *Cr*-P*psbA* were amplified from the Chlamydomonas 4A+ genomic DNA. Those elements were constructed into pCG2-Nluc vector, resulting in eight vector combinations, *Cr*-P*rbcL/Cr*-T*psbA*, *Cr*-P*psbA/Cr*-T*psbA*, *Nt*-P*rbcL/Cr*-T*psbA*, *Nt*-P*psbA/Cr*-T*psbA*, *Cr*-P*psbD/Nt*-T*rbcL*, *Cr*-P*psbD/Nt-*T*rps16*, *Nt*-P*psbD/Nt*-T*rbcL* and *Nt*-P*psbD/Nt*-T*rps16*. The 5’ flanking homologous arm of the pCG2 vector was integrated into the Chlamydomonas chloroplast genome from 156,533 to 157,648, while the 3’ flanking homologous arm spanned from position 159,278 to 161,562 (Chen et al., 2013). Primers used for PCR amplification of promoters and terminators were listed in Table S1.

Chlamydomonas IEE vectors utilized intercistronic regions from the *psbN-psbH* (Cr-IEE2) and *tscA-chlN* (Cr-IEE5) operons, as identified previously (Macedo-Osorio et al., 2018). The Chlamydomonas IEE2 and IEE5 were amplified from the Chlamydomonas genomic DNA and inserted into the pCG2-Cr-IEE vector. The upstream and downstream fragments of IEE consisted of *cbbL* and *Nluc* genes, controlled by Chlamydomonas P*psbD* promoter and T*psbA* terminator. The *cbbL* gene was derived from the Rubisco large subunit of *Thiobacillus neapolitanus* and was codon-optimized for gene expression in *Chlamydomonas reinhardtii* chloroplast. Tobacco IEE was derived from the *psbT*-*psbH* intercistronic region (Zhou et al., 2007), with the SD sequence (GGGAGGGATTT) from tobacco *RbcL* gene. In the tobacco synthetic operon, the *cbbL* and *Nluc* genes flanked the tobacco IEE, with the expression cassette under the control of Chlamydomonas P*psbD* promoter and T*psbA* terminator.

The pCG2-VOR-Rep-VOR vector was constructed based on the pCG2-Nluc vector using Rep amplification primers. Furthermore, two repeating VOR sequences were constructed at both ends of the expression cassettes. The Rep protein from beet curly top geminivirus (BCTV), was synthesized following the codon preference of Chlamydomonas chloroplast. The pVOR-cbbS-VOR vector was generated by amplifying and inserting the cbbS and aadA expression units into an intermediate vector, devoid of 5' and 3' homologous arms. The promoters and terminators utilized are from Chlamydomonas genes.

**Chloroplast transformation**

Procedures of the Chlamydomonas chloroplast transformation were based on an established biolistic method (Boynton & Gillham, 1993). Briefly, a suspension of 250 mL Chlamydomonas cells was prepared and spread onto three TAP agar plates. Plasmids (5-10 µg) were coated with 50 μL resuspended (~3 mg) gold particles, along with 50 μL of 2.5 M CaCl_2_ and 20 μL of 0.1 M spermidine. The gene gun (PDS-1000/He Biolistic Particle Delivery System; Bio-Rad, Hercules, CA, USA) was pressurized to approximately 1,500 psi for bombardment. Following transformation, the resulting transformants were screened on TAP agar plates supplemented with 100 µg/mL spectinomycin. A concentration of 200 µg/mL spectinomycin was used to achieve homoplasmy. The vectors pCG2-Nluc, pCG2-Cr-IEE, pCG2-Nt-IEE and pCG2-VOR-Rep-VOR were used for transformation into 4A+ strain. The pVOR-cbbS-VOR vector was used for transformation into pRbcL-Rep strain, which contains RbcL and Rep expression units.

**RNA extraction and quantitative real-time PCR**

Total RNA was extracted from pCG2-Nluc, pCG2-cbbL, pCG2-Cr-IEE and pCG2-Nt-IEE transgenic lines using the TransZol plant kit (TransGen Biotech, Beijing, China). RNA was used to synthesize the first-strand cDNA according to the instructions of the PerfectStart^®^ Uni RT&qPCR Kit (TransGen Biotech, Beijing, China). The qRT-PCR was performed using TransStart^®^Tip Green qPCR SuperMix (TransGen Biotech, Beijing, China) on Bio-Rad CFX96 Real-Time PCR Detection System. The *CBLP* gene was used as an endogenous control. The 2^−ΔΔCt^ method was used to estimate the relative expression level of the target gene. All reactions were performed with three biological replicates. The qRT-PCR primes used in this study were listed in Table S1. Significant differences were analyzed using one-way ANOVA followed by Tukey's multiple comparisons test (P < 0.05).

**Protein extraction and immunoblot analysis**

The extraction of Chlamydomonas total protein was carried out using the methanol-chloroform method (Duanmu et al., 2013). Around thirty micrograms of total protein were isolated through 10% SDS-polyacrylamide gel electrophoresis and subsequently transferred to PVDF membrane for immunoblotting. The anti-STII antibody was obtained from PhytoAB (PHY5022).

**Analysis of Nluc** **luciferase activity**

NanoLuc (Nluc), a luciferase derived from the deep sea shrimp *Oplophorus gracilirostris* (Hall et al., 2012), was synthesized based on the codon usage preference of Chlamydomonas chloroplast genome. The luminescence signal of Nluc was assessed using the Nano-Glo^®^ Luciferase Assay System (Promega, N1120). To prepare the luciferase assay reagent, one volume of assay substrate was mixed with 50 volumes of assay buffer. Equal volume of 50 µL luciferase assay reagent and 50 µL Chlamydomonas cells (~1×10^6^ cells/mL) were mixed in a 96-well plate, incubated for a minimum of 3 minutes, and then the signal was captured using a CCD imaging apparatus (4600 series fully automated chemiluminescence imaging analysis system; Tanon, Shanghai, China).

**References**

Boynton, J.E. & Gillham, N.W. (1993) Chloroplast transformation in Chlamydomonas. *Methods Enzymol*, 217, 510-536.

Chen, W.N., Demurtas, O.C., Massa, S., Ferrante, P., Venuti, A., Franconi, R. et al. (2013) A Chlamydomonas-Derived Human Papillomavirus 16 E7 Vaccine Induces Specific Tumor Protection. *PLoS One*, 8, e61473.

Duanmu, D., Casero, D., Dent, R.M., Gallaher, S., Yang, W., Rockwell, N.C. et al. (2013) Retrograde bilin signaling enables Chlamydomonas greening and phototrophic survival. *Proceedings of the National Academy of Sciences*, 110, 3621-3626.

Hall, M.P., Unch, J., Binkowski, B.F., Valley, M.P., Butler, B.L., Wood, M.G. et al. (2012) Engineered Luciferase Reporter from a Deep Sea Shrimp Utilizing a Novel Imidazopyrazinone Substrate. *ACS Chemical Biology*, 7, 1848-1857.

Macedo-Osorio, K.S., Perez-Espana, V.H., Garibay-Orijel, C., Guzman-Zapata, D., Duran-Figueroa, N.V. & Badillo-Corona, J.A. (2018) Intercistronic expression elements (IEE) from the chloroplast of Chlamydomonas reinhardtii can be used for the expression of foreign genes in synthetic operons. *Plant Molecular Biology*, 98, 303-317.

Zhou, F., Karcher, D. & Bock, R. (2007) Identification of a plastid intercistronic expression element (IEE) facilitating the expression of stable translatable monocistronic mRNAs from operons. *Plant Journal*, 52, 961-972.
